# Hydrolysis of Cellulose by a Mesoporous Carbon-Fe_2_(SO_4_)_3_/γ-Fe_2_O_3_ Nanoparticle-Based Solid Acid Catalyst

**DOI:** 10.1038/srep20327

**Published:** 2016-02-09

**Authors:** Daizo Yamaguchi, Koki Watanabe, Shinya Fukumi

**Affiliations:** 1 Department of Mechanical Engineering, National Institute of Technology, Tsuyama College, 624-1 Numa, Tsuyama-City, Okayama 708-8509, Japan

## Abstract

Carbon-based solid acid catalysts have shown significant potential in a wide range of applications, and they have been successfully synthesized using simple processes. Magnetically separable mesoporous carbon composites also have enormous potential, especially in separation and adsorption technology. However, existing techniques have been unable to produce a magnetically separable mesoporous solid acid catalyst because no suitable precursors have been identified. Herein we describe a magnetically separable, mesoporous solid acid catalyst synthesized from a newly developed mesoporous carbon-γ-Fe_2_O_3_ nanoparticle composite. This material exhibits an equivalent acid density and catalytic activity in the hydrolysis of microcrystalline cellulose, to that of the cellulose-derived conventional catalyst. Since it is magnetically separable, this material can be readily recovered and reused, potentially reducing the environmental impact of industrial processes to which it is applied.

A highly active, carbon-based solid acid catalyst has been reported^1^,^2^,^3^. This material is a solid Brønsted acid composed of randomly distributed graphene sheets bearing SO_3_H-, COOH- and phenolic OH- functional groups. Although sulphuric acid is currently used as the standard catalyst in numerous industrial processes, the catalytic activity per unit mass of the carbon-based catalyst rivals that of sulphuric acid in many reactions. In addition, given the low effective acid density (4.3 mmol g^−1^) and low surface area (2–20 m^2^ g^−1^) of this catalyst, the reactivity of its Brønsted acid sites is at least 10 times greater than that of sulphuric acid^2^,^3^. This catalyst performs as well as mineral acid catalysts in the hydrolysis of both pure crystalline cellulose and natural lignocellulosic substrates into water-soluble β-1,4 glucan with subsequent hydrolysis to glucose^3^,^4^,^5^. The catalyst itself is readily prepared by incomplete carbonization of sulphopolycyclic aromatic hydrocarbons or by sulphonation of incompletely carbonized organic compounds^2^. While it is true that carbon-based solid acid catalysts can also be synthesized by sulphonation of carbon nanotubes^6^ or activated carbon materials^7^, the density of SO_3_H groups in these materials is much lower than in the carbon-based catalysts.

Magnetically separable mesoporous carbon materials have enormous potential for applications as catalytic supports, in separation technology and for the adsorption of biomolecules^8^,^9^,^10^,^11^,^12^,^13^,^14^. Despite this, a solid acid catalyst which is also a magnetically separable material has not previously been developed, since no suitable precursor compounds were known. Much effort has been expended in attempts to prevent the leaching and oxidation of deposited metal nanoparticles during complicated multi-step synthetic procedures to produce a precursor, a magnetic mesoporous carbon material. The necessity of using a complicated synthetic procedure involving an expensive carbon source and high temperatures to generate sp^3^ carbon structures in graphite has discouraged the production of magnetically separable solid acid catalysts.

Recently, a new, simple and robust method for the preparation of a mesoporous carbon-γ-Fe_2_O_3_ nanoparticle composite (MCNC) has been reported^15^. This material can be directly synthesized in a one-pot, non-templating and self-sustaining reaction using carboxymethylcellulose as an inexpensive carbon source. Herein, a mesoporous magnetically separable MCNC-based solid acid catalyst (MCNC-SA) bearing SO_3_H, COOH, and OH surface functional groups and containing Fe_2_(SO_4_)_3_/γ-Fe_2_O_3_ nanoparticles in its carbon matrix is reported. This new catalyst offers significant performance advantages since its concentration of acid density and its catalytic activity, in the hydrolysis of microcrystalline cellulose, is equal to that of the cellulose-derived conventional catalyst. This paper discusses the measurement and analysis of the catalytic properties and activities of this novel material.

## Results

### Structure and Morphology

**Figure 1:**
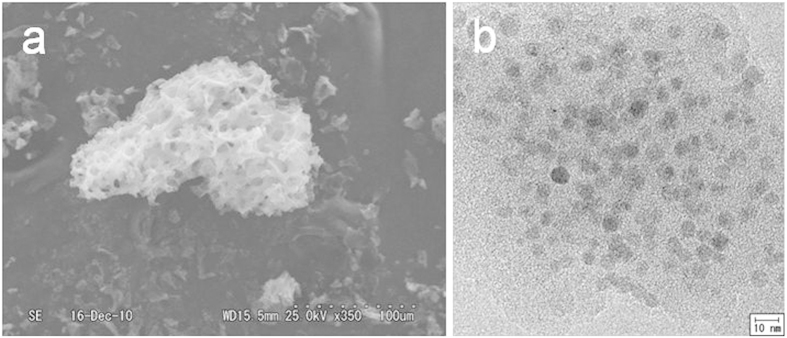
Morphologies of MCNC-SA (concentration of iron(III) nitrate enneahydrate solution: 5.0 g L^−1^). (**a**) SEM image and (**b**) TEM image.

As might be expected, the structure of MCNC-SA is very similar to that of the MCNC from which it is derived^15^. The particle size ranges (Fig. 1a) and Brauner-Emmett-Teller (BET) surface areas of MCNC-SA are estimated to be 2–200 μm and 17–130 m^2^ g^−1^ (Table S1), respectively. The data in Table S1 demonstrate that the BET surface areas of the materials increase with increasing concentration of the iron(III) nitrate enneahydrate solution used in their synthesis, as with the MCNC. The N_2_ adsorption-desorption isotherms of the material resemble those of the MCNC (Figure S1a), suggesting that mesopores and micropores still coexist^15^,^16^. The total pore volume and the average size of ideal cylindrical pores of MCNC-SA, based on the BJH (Barrett–Joyner–Halenda) method^17^, are in the ranges of 0.06–0.22 cm^3^ g^−1^ and 7–14 nm, respectively (Figure S1b, Table S1), slightly different from the base MCNC. Figure 1b presents a transmission electronic microscopy (TEM) image of the sample. Analysis of the sample via X-ray photoelectron spectroscopy (XPS) and elemental analysis also revealed the presence of Fe, O, Na, C and S (Table S2). The material includes distributed iron oxide and/or iron sulphite nanoparticles (5–10 nm in size, 1.4–6.3% in weight percent) within its carbon matrices. In general, iron oxide nanoparticles smaller than approximately 20 nm display superparamagnetic behavior at room temperature (the critical sizes for maghemite and magnetite are 10 and 6 nm, respectively)^18^. Unfortunately, it was not possible to determine a blocking temperature for the sample in measurements using a superconductive quantum interference device (SQUID) (as shown in Figure S2). Therefore, it seems that most of the iron oxide particles in the materials synthesized in this work do not exhibit superparamagnetic behavior. Figure S3, TEM images of MCNC-SA, shows that the average particle size in the MCNC-SA carbon matrix was reduced as the concentration of the iron(III) nitrate enneahydrate solution was increased.

Figures S4a and b provide the powder X-ray diffraction (XRD) pattern and Raman spectrum of MCNC-SA, which are very similar to those of MCNC^15^. Obvious peaks characteristic of Fe_2_(SO_4_)_3_ (JCPDS card No. 33–0679) are absent in the MCNC-SA, although it is possible that the broad peak in the range 15°–30° may be partly attributed to Fe_2_(SO_4_)_3_ (overlapped with the peak indicating carbon with a very low degree of graphitization^19^). In the Raman spectrum of MCNC-SA (Figures S4b), the average graphene dimension in the carbon is approximately 1 nm^3^,^15^,^20^.

**Figure 2:**
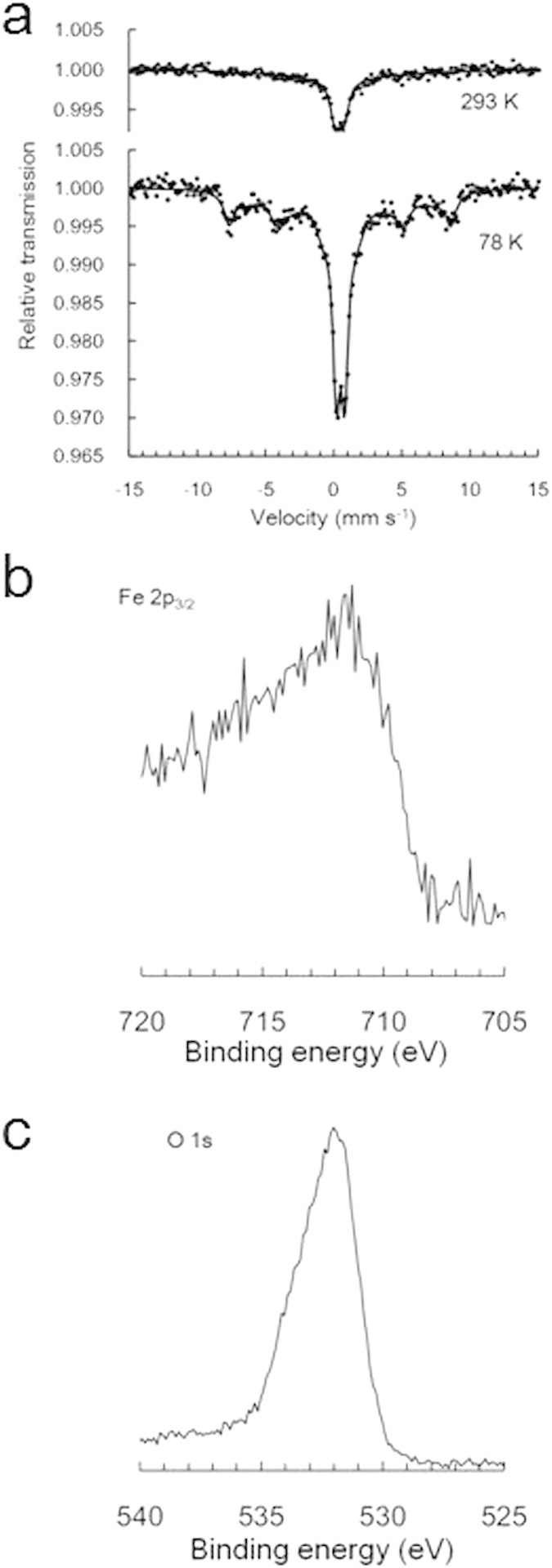
Characterization of MCNC-SA (concentration of iron(III) nitrate enneahydrate solution: 5.0 g L^−1^). (**a**) Mössbauer spectrum at 293 K and 78 K, (**b**) Fe 2p_3/2_ and (**c**) O 1s XPS spectra.

Figure 2a shows the Mössbauer spectra of MCNC-SA at 293 K and 78 K. At room temperature, the spectrum is best described as two doublets, although this is very similar to that of MCNC^15^. The summary of Mössbauer data is shown in Table S3. These data indicate that the majority of the nanoparticles are paramagnetic^15^. The spectrum at liquid nitrogen temperatures (78 K) exhibits a strong doublet and two sextets, although the paramagnetic component remains paramagnetic in MCNC-SA. The strong doublet in this spectrum is the evidence for the presence of a relatively large amount of paramagnetic Fe^3+^ in MCNC-SA^15^.

The magnetic field distribution was analyzed under the assumption that the paramagnetic component has a quadrupole shift doublet. Although local maximal values of the magnetic field were observed at 510 and 150 kOe, the mode value of the internal magnetic field distribution was 20–30 kOe (Figure S5), and some components were not well fit by the model. Therefore, the results suggest that, although MCNC-SA contains γ-Fe_2_O_3_, this is not the only component. Additional analysis via XPS demonstrated the presence of iron(III) sulphate (Fe_2_(SO_4_)_3_) in MCNC-SA (Fig. 2b). The presence of Fe_2_(SO_4_)_3_ was confirmed by a narrow range analysis of Fe 2p_3/2_ (713.4 eV). The Mössbauer effect parameters of Fe_2_(SO_4_)_3_ are as follows: isomer shift of 0.39 mm s^−1^, quadrupole shift of 0.60 mm s^−1^ at room temperature^21^ with an indicative sextet of 550 ± 10 kOe at 1.8 K^22^. Moreover, the Néel temperature of Fe_2_(SO_4_)_3_ is approximately 30 K^23^,^24^,^25^. Consequently, the results show that MCNC-SA contains both Fe_2_(SO_4_)_3_ and γ-Fe_2_O_3_, providing evidence that the majority of the γ-Fe_2_O_3_ particles that were exposed on the MCNC^15^ surface were converted to Fe_2_(SO_4_)_3_ during the sulphonation process.

In the O 1s spectrum (Fig. 2c), the peak is somewhat asymmetric because of the presence of oxygen-containing impurities in the carbon framework. The reported values for the O 1s binding energies of α-Fe_2_O_3_ and Fe_3_O_4_ are identical at approximately 530.0 eV, slightly below that of γ-Fe_2_O_3_ (530.6 eV)^26^. The value of 531.7 eV obtained in the O 1s spectra is a good match with the expected value for γ-Fe_2_O_3_, and the lack of a shoulder peak on the lower energy side establishes the absence of Fe_3_O_4_ and α-Fe_2_O_3_. In addition, the presence of a distinct shoulder at 533.5 eV may be attributed to the sulphite group in Fe_2_(SO_4_)_3_. XPS analysis also confirmed the presence of peaks associated with SO_3_H groups (at 168.4 eV)^2^ and Fe_2_(SO_4_)_3_ (at 169.6 eV) (see the S 2p spectra in Figure S6a).

The ^13^C dipolar decoupling magic angle spinning nuclear magnetic resonance (^13^C DD/MAS NMR) spectrum of MCNC-SA is shown in Figure S6b. This spectrum contains a number of readily identifiable peaks at 128 (polycyclic aromatic carbons), 139 (Ar–SO_3_H), 156 (phenolic OH) and 185 (COOH) ppm^1^,^2^,^3^. Figure S6c provides the Fourier transform infrared (FTIR) spectrum for MCNC-SA, in which the bands at 1041 (S=O stretching), 1200 (SO_3_-stretching) and 1382 cm^−1^ (O=S=O stretching in SO_3_H), and the broad band at 2000–2500 cm^−1^ indicate the presence of SO_3_H groups on the MCNC-SA^3^,^27^. In the ^13^C DD/MAS NMR spectrum, there are no signals between 0 and 100 ppm. However, the FTIR data show small bands characteristic of aliphatic C-H bands at 2850–3000 cm^−1^ (sp^3^ C-H stretching). We therefore conclude that the materials contain sp^3^ carbon atoms.

### Magnetic and catalytic properties

**Figure 3:**
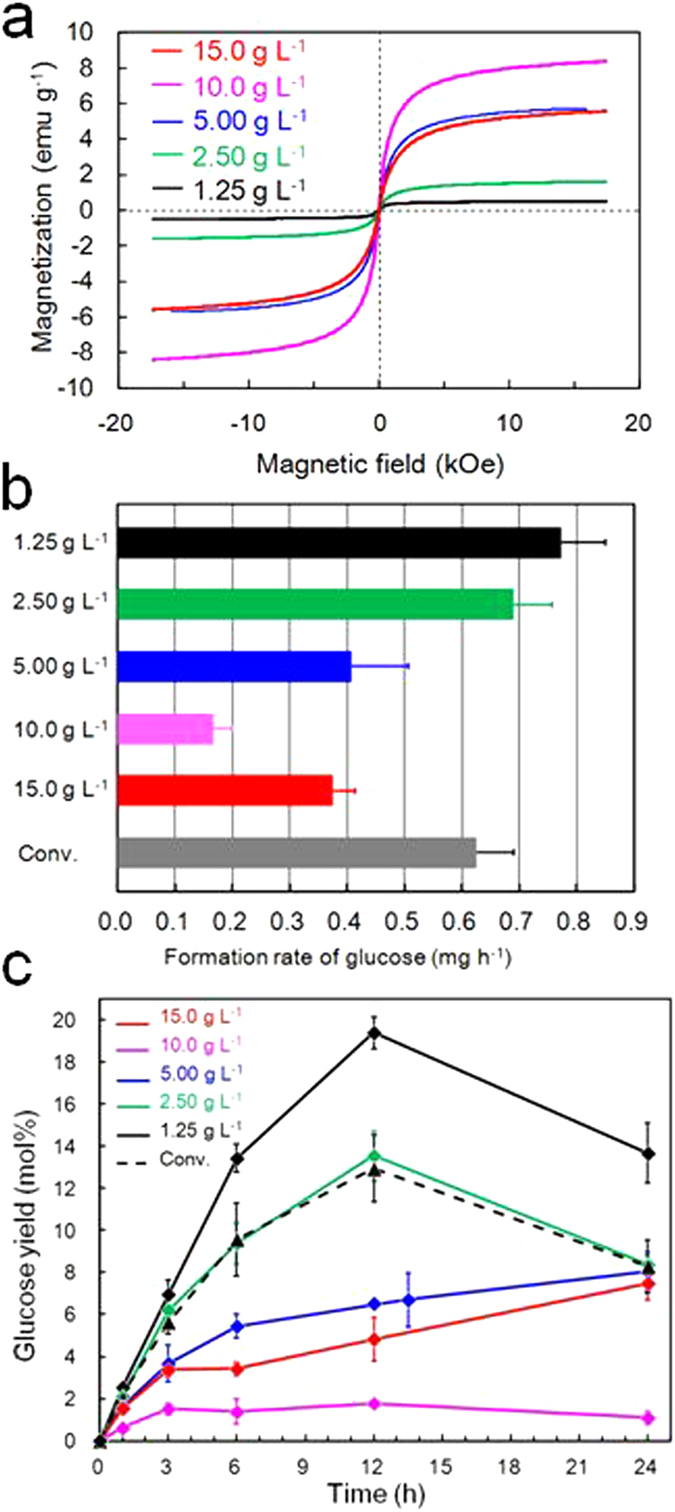
Magnetic and catalytic activity of MCNC-SA (concentration of iron(III) nitrate enneahydrate solution: black 1.25 g L^−1^; green, 2.50 g L^−1^; blue, 5.00 g L^−1^; pink, 10.0 g L^−1^; red, 15.0 g L^−1^). (**a**) hysteresis curves of MCNC-SA at room temperature, (**b**) glucose formation rates during the hydrolysis of cellulose with MCNC-SA or a conventional solid acid catalyst (grey). Reaction conditions: catalyst, 30 mg; cellulose, 30 mg; water, 0.3 g; reaction temperature, 120 °C; time, 3 h, and (**c**) the time course of glucose yield during the hydrolysis of cellulose using either MCNC-SA or a conventional solid acid catalyst^3^ (broken line). Error bars denote standard error. Reaction conditions: MCNC-SA, 30 mg; cellulose, 30 mg; water, 0.3 g; reaction temperature, 120 °C.

The magnetic properties of the obtained materials at room temperature are provided in Fig. 3a. These curves are nearly superimposable as the field is cycled between -17 and 17 kOe (Table S4). The saturation magnetization (*M*_s_) values are seen to increase with increasing concentrations of the iron nitrate solution, reaching a maximum at 10 g L^−1^. The *M*_s_ values of MCNC were seen to increase with increasing concentrations of the iron nitrate solution^15^; however, these values are decreased significantly (by 50–90%) following sulphonation to produce MCNC-SA. This is attributable to the transformation of γ-Fe_2_O_3_ to Fe_2_(SO_4_)_3_ and the concurrent dissolution of iron oxide. These data indicate that the two processes of creating the solid acid catalyst and of adding magnetic properties conflict with one another, such that it would be impossible to synthesize MCNC-SA, which exhibits an *M*s value of 8.38 emu g^−1^ and is also a carbon-based solid acid catalyst, without using MCNC as a precursor. If MCNC-SA is to be prepared with sufficient magnetic properties that it may be magnetically separated from the reaction solution, the lower limit for the concentration of the iron nitrate solution applied during synthesis appears to be 1.25 g L^−1^. The optimal iron nitrate concentration for MCNC-SA from a catalytic perspective is revealed by the catalytic properties described below.

Catalytic properties were evaluated by Boehm titration with elemental sulphur analysis^28^ (Table 1). The SO_3_H density of the most carbon rich condition (iron nitrate concentration: 1.25 g L^−1^) showed a maximum of 1.62 mmol g^−1^, which is an equivalent acid density to that of the cellulose-derived conventional catalyst^4^. Sulphur percentage was proportionate to carbon percentage. As demonstrated in the cellulose hydrolysis experiment (Fig. 3b), catalytic activity was determined by SO_3_H density, because the acidity of COOH and OH groups is negligibly weak^28^. The rate of glucose formation decreases with increasing concentrations of the iron nitrate solution, reaching a minimum at 10 g L^−1^ before increasing again at 15 g L^−1^.

Figure 3c provides plots of the time course of the glucose yield as a function of the catalyst applied. Our analysis also determined that glucose was not the sole hydrolysis product since, during the course of the hydrolysis reactions, various by-products were identified. These included xylose, which results from impurities in the initial cellulose powder, levoglucosan, 5-hydroxymethyl-2-furaldehyde, formic acid, and levulinic acid. During hydrolysis with the MCNC-SA synthesized using the 1.25 g L^−1^ iron nitrate solution, by-products accounted for 0.97, 1.72, 2.55, 5.17, and 5.96 mol% of the total yield following 1, 3, 6, 12, and 24 hours of reaction time, respectively. These elevated levels of by-product generation can be attributed to the high temperature applied during the hydrolysis reaction^5^.

## Discussion

Figure 3c shows that, during the first 3 hours of hydrolysis, the glucose formation rates are essentially equal for the 1.25 and 2.50 g L^−1^ catalyst specimens and the conventional cellulose-derived catalyst, and that all three also exhibit roughly constant hydrolysis rates over this period. After 6 hours, however, all three show pronounced decreases in their reaction rates, which may be attributed either to a shortage of the water required for the reaction or to blocking of acid sites by the reaction products. The deleterious effect of decreasing water concentrations in the reaction mixture is due to the basic nature of solid acid-catalyzed hydrolysis. In the early stages of the reaction, there is typically a significant excess of water, which is advantageous in terms of the hydrolysis rate, both in terms of reaction kinetics and equilibrium. As the reaction progresses, however, water is consumed and, while this markedly increases the acidity of the solid acid catalyst, it also decreases the hydrolysis rate.

Figure S7a shows repeated experiments in the hydrolysis of cellobiose (water-soluble *β*-1,4-glucan) using MCNC-SA (1.25 g L^−1^). Since cello-oligosaccharides could not be completely removed from the reaction mixture when microcrystalline cellulose was used for the experiment, cellobiose has been used as a substrate to verify the efficiency of the reaction. Cellulose is a water-insoluble substrate of long-chain *β*-1,4 glucan composed of glucose monomers linked by *β*-1,4 glycosidic bonds. Therefore, the catalyst is expected to convert cellulose into water-soluble saccharides by the hydrolysis of the *β*-1,4 glycosidic bonds and decomposition of the hydrogen bonds linking *β*-1,4 glucan chains. Cellobiose consists of two *β*-glucose molecules linked by a *β*-1,4 bond, allowing a simple test of catalytic hydrolysis. The catalyst retained its activity through 5 reuses, although the activity decreased to one quarter after the first time, and then stabilised at the lower level. Remarkably, reactivation after the fifth use restored the catalytic activity for the sixth use, and the level of activity displayed good stability on a seventh use. As result of the equilibrium between sulphoaromatic compounds and aromatic hydrocarbons, SO_3_H bond to aromatic hydrocarbons is generally subject to leaching in the presence of water^4^. Siril *et al.* have reported that the aromatic carbon-SO_3_H bonds in sulphoaromatic compounds bearing electron-withdrawing functional groups such as halogens are more stable as a result of the increased electron density between carbon and sulphur atoms afforded by the electron-withdrawing functional group^29^. Since the carbon with a very low degree of graphitization in this novel material possesses SO_3_H and COOH groups, it is also expected that electron-withdrawing functional groups (COOH) will increase the electron density between the carbon and sulphur atoms. Nevertheless, SO_3_H leaching has occurred. The presence of iron oxide may also influence the destabilization of surface functional groups.

Normally, since anhydrous Fe_2_(SO_4_)_3_ is slightly soluble in water, we would expect some leaching of this compound from the catalyst matrix during the hydrolysis reaction. However, this is unlikely to occur when using MCNC-SA since, following sulphonation, the catalytic material was rinsed with hot distilled water until impurities such as sulphate were no longer detected in the wash water. In fact, XPS analysis revealed the presence of Fe_2_(SO_4_)_3_ after the catalytic reaction (Figure S7b, c). For this reason, there appears to have been no loss of Fe_2_(SO_4_)_3_ from the catalyst, and tests showed that the magnetic properties of the recovered MCNC-SA were not reduced subsequent to the hydrolysis reaction.

In conclusion, a magnetically separable mesoporous carbon-based solid acid catalyst was prepared. This catalyst demonstrated an equivalent acid density and catalytic activity in the hydrolysis of microcrystalline cellulose, to that of a cellulose-derived conventional catalyst. The catalyst produced with an iron nitrate concentration of 1.25 g L^−1^ demonstrates fifty percent greater catalytic activity than the conventional catalyst over a 12 hour reaction. This solid acid was synthesized from a newly developed mesoporous carbon-γ-Fe_2_O_3_ nanoparticle composite precursor (MCNC), which was, in turn, made by reacting CMC with iron(III) nitrate enneahydrate solution^15^. This material, based on carbon with a very low degree of graphitization bearing SO_3_H, COOH and OH groups, and containing Fe_2_(SO_4_)_3_/γ-Fe_2_O_3_ nanoparticles in its carbon matrix, was shown to function as a highly active catalyst for the direct hydrolysis of cellulose, an important step both in the production of biofuels and also in the synthesis of chemical products from non-food plants. This catalyst does not contain heavy or rare metals and can easily be recovered from the reaction medium, thus reducing the environmental impact of any industrial processes in which it is employed.

## Methods

### Synthesis

The MCNC powder, described elsewhere^15^, (3 g) was boiled in a mixture of fuming sulphuric acid (50 mL, 20 wt% SO_3_) and concentrated sulphuric acid (50 mL, 98 wt% H_2_SO_4_) at 80 °C. After heating for 10 h and then cooling to room temperature, 3000 cm^3^ of hot distilled water was used to rinse the mixture until impurities such as sulphate ions were no longer detected in the wash water^3^.

### Characterization

The morphologies of the synthesized materials were determined by scanning electron microscopy (SEM, Hitachi High-Technologies S-3000N), nitrogen adsorption and desorption isotherms (including BET surface area measurements, Quantachrome Instruments NOVA 4200e) and FE-TEM/EDS (Topcon Technohouse EM002BF). Structural information for the materials was obtained by XRD (CuKα, λ = 1.54056 Å, 40 kV, 200 mA (focusing method), Rigaku RINT1500), Raman spectroscopy (Jobin Yvon T-64000), ^57^Fe Mössbauer spectroscopy (Toray Research Center), XPS (AlKα, 1486.6 eV, ULVAC-PHI Quantera SXM), FTIR with an attenuated total reflectance (ATR, PRO450-S) unit at room temperature (JASCO FT/IR-6300), and ^13^C DD/MAS NMR (JEOL JNM-ECX400) at room temperature. The magic angle was controlled at a 15 kHz sample tube (4 mm) rotation rate and the background signal was acquired using KBr under the same conditions. Mössbauer spectra were recorded on a Wissel spectrometer in the constant acceleration mode (transmission method), using a ^57^Co(Rh) source (1.85 GBq). Samples for Mössbauer analysis were prepared by mixing 80 mg of material and 10 mg of high-purity polyethylene, followed by pressing at 14.7 MPa. The elemental compositions of the samples were determined using two methods. Precise values for C, H, N and S were obtained using an elemental analyzer (J-Science Lab. JM-10), while values for O, Na and Fe were estimated from XPS analysis. The elements measured as ash in elemental analysis of the novel material are iron oxide and sodium oxide. Since sodium oxide is neutralized by the addition of sulphuric acid, the ash of the catalyst is almost entirely Fe_2_O_3_. The iron content has been calculated from this datum. Concentrations of functional groups were determined by Boehm titration and elemental sulphur analysis^28^. In our case, as the colour of reagents cannot be determined by visual inspection on these black carbon materials, and the magnetic properties of the material rule out ^31^P MAS NMR examination. Magnetization measurements of the material were performed using VSM (Toei Industry VSM-15) at room temperature (25 °C) and SQUID (Quantum Design SQUID magnetometer MPMS3) at 5 K to 300K (1000 Oe).

### Acid-catalyzed reaction

The catalytic performance of this material was studied by monitoring the hydrolysis of microcrystalline cellulose (Avicel^®^, particle size 20–100 μm; crystallinity 80%; degree of polymerization 200–300) and cellobiose (for the repeated experiments^4^), carried out in 12 cm^3^ vials equipped with a stir bar and seal cap. The catalytic activities of both MCNC-SA and a conventional solid acid catalyst were investigated using the following conditions: catalyst, 30 mg; cellulose, 30 mg; water, 0.3 g; reaction temperature, 120 °C. The hydrolysis reaction within each vial was initiated by placing the vial in a preheated oil bath. Following hydrolysis, an aliquot of the supernatant solution was readily separated using a neodymium magnet and was subsequently passed through a 0.45 μm filter membrane and analyzed via high performance liquid chromatograph (Shimadzu RID-10A HPLC; column temperature, 20 °C; mobile phase, 5 mM H_2_SO_4_ at 0.5 ml min^−1^). Repeated experiment was performed as described elsewhere^4^. Reactivation was conducted in the same manner^3^, as follows: 0.27 g of used MCNC-SA (1.25 g L^−1^) was boiled in a mixture of 10 mL fuming sulphuric acid and 10 mL concentrated sulphuric acid at 80 °C for 10 h. 3000 cm^3^ of hot distilled water was used to rinse the mixture.

## Additional Information

**How to cite this article**: Yamaguchi, D. *et al.* Hydrolysis of Cellulose by a Mesoporous Carbon-Fe_2_(SO_4_)_3_/γ-Fe_2_O_3_ Nanoparticle-Based Solid Acid Catalyst. *Sci. Rep.* **6**, 20327; doi: 10.1038/srep20327 (2016).

## Supplementary Material

Supplementary Information

## Figures and Tables

**Table 1:** Concentration of acid group of MCNC-SA.

Samples^a^(with iron nitrate concentration)	−SO_3_H(mmol g^−1^)	−COOH(mmol g^−1^)	−OH(mmol g^−1^)	H/C	S/C
1.25 g L^−1^	1.62	2.04	0.09	0.48	0.032
2.50 g L^−1^	1.51	1.67	0.16	0.50	0.031
5.00 g L^−1^	1.45	1.52	0.04	0.41	0.032
10.0 g L^−1^	1.38	1.75	0.01	0.43	0.032
15.0 g L^−1^	1.46	1.82	0.21	0.51	0.032

^a^Determined by Boehm titration with elemental sulphur analysis^28^.
