# Supplementary material for: Hydrolysis of Cellulose by a Mesoporous Carbon-Fe2(SO4)3/γ-Fe2O3 Nanoparticle-Based Solid Acid Catalyst
Source: Sci Rep. 2016 Feb 9;6:20327. doi: 10.1038/srep20327 (PMC4746576; doi:10.1038/srep20327)
Supplement: Supplementary Information [file srep20327-s1.doc]

Supplementary information

Hydrolysis of Cellulose by a Mesoporous Carbon-Fe_2_(SO_4_)_3_/γ-Fe_2_O_3_ Nanoparticle-Based Solid Acid Catalyst

Daizo Yamaguchi^1*^, Koki Watanabe^1^, Shinya Fukumi^1^

^1^Department of Mechanical Engineering, National Institute of Technology, Tsuyama College, 624-1 Numa, Tsuyama-City, Okayama 708-8509, Japan

^*^Correspondence and requests for materials should be addressed to D.Y. (tnt_yama@tsuyama-ct.ac.jp)

Table of Contents

Table S1. BET surface areas, total pore volumes and average pore sizes S3

Figure S1. Pore size distribution of the sample 　　 S3

Table S2. Sample compositions S4

Figure S2. Zero field cooled and field cooled magnetization for MCNC-SA S4

Figure S3. Iron oxide nanoparticle sizes S5

Figure S4. XRD and Raman spectra S5

Table S3. Mössbauer effect parameters S6

Figure S5. Internal magnetic field distribution S6

Figure S6. Surface functional groups S7

Table S4. Magnetic remanence, saturation magnetizations and coercivity S8

Figure S7. Repeated hydrolysis of cellobiose S8

Legend

MCNC: mesoporous carbon-γ-Fe_2_O_3_ nanoparticle composite

MCNC-SA: MCNC-based solid acid catalyst

Table S1 | Brauner-Emmett-Teller (BET) surface areas, total pore volumes, and average pore sizes of MCNC-SA samples synthesized using different iron nitrate concentrations.

| Samples  (with iron nitrate  concentration) | BET specific surface area (m^2^ g^-1^) | Total pore volume (cm^3^ g^-1^) | Average pore size (nm) |
| --- | --- | --- | --- |
| 1.25 g L^-1^ | 17.4 | 0.060 | 13.90 |
| 2.50 g L^-1^ | 39.1 | 0.103 | 10.51 |
| 5.00 g L^-1^ | 65.6 | 0.106 | 6.44 |
| 10.0 g L^-1^ | 114.4 | 0.177 | 6.20 |
| 15.0 g L^-1^ | 129.1 | 0.218 | 6.76 |

**
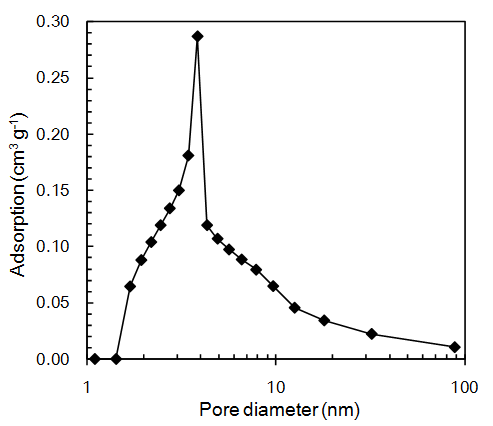

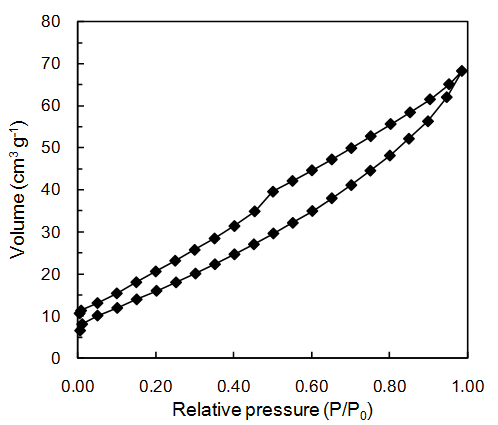
**

a

b

Figure S1 | Pore size distributions of MCNC-SA (concentration of iron(III) nitrate enneahydrate solution: 5.0 g L^-1^). (a) Nitrogen adsorption-desorption isotherms. (b) Pore size distribution obtained from desorption isotherms as calculated by the BJH (Barret-Joyner-Halenda) method.

Table S2 | Compositions of MCNC-SA.

| Sample ^a^  (with iron nitrate  concentration) | C (1s) | N (1s) | O (1s) | Na (1s) | S (2p) | Fe (2p_3/2_) |
| --- | --- | --- | --- | --- | --- | --- |
| 5.0 g L^-1^ | 71.7 | 1.3 | 23.5 | 0.2 | 2.4 | 0.9 |

1. Elemental compositions by XPS analysis, in atomic percent.

| Samples ^b^  (with iron nitrate concentration) | C | H | N | S | Fe ^c^ |
| --- | --- | --- | --- | --- | --- |
| 1.25 g L^-1^ | 60.22 | 2.40 | 1.03 | 5.18 | 1.40 |
| 2.50 g L^-1^ | 58.53 | 2.44 | 0.89 | 4.83 | — |
| 5.00 g L^-1^ | 54.18 | 1.85 | 0.66 | 4.64 | 6.21 |
| 10.0 g L^-1^ | 52.09 | 1.86 | 0.69 | 4.41 | — |
| 15.0 g L^-1^ | 55.06 | 2.34 | 0.84 | 4.69 | 6.33 |

1. Compositions determined by elemental analyzer, in weight percent.
2. Calculated from ash (Fe_2_O_3_) weight percent.


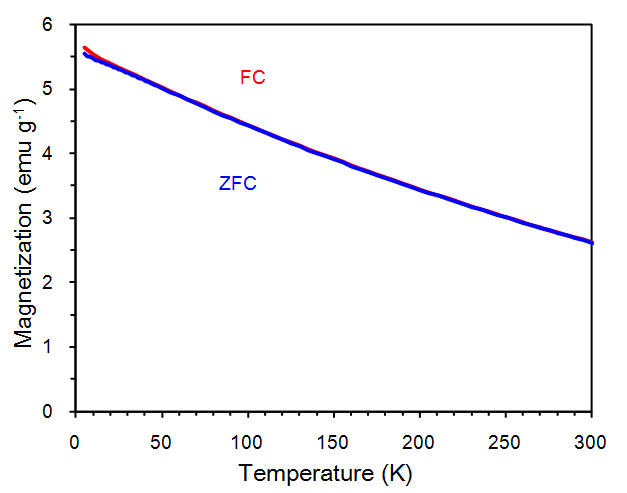


Figure S2 | Zero field cooled (ZFC) and field cooled (FC) magnetization for MCNC-SA (concentration of iron(III) nitrate enneahydrate solution: 5.0 g L^-1^).


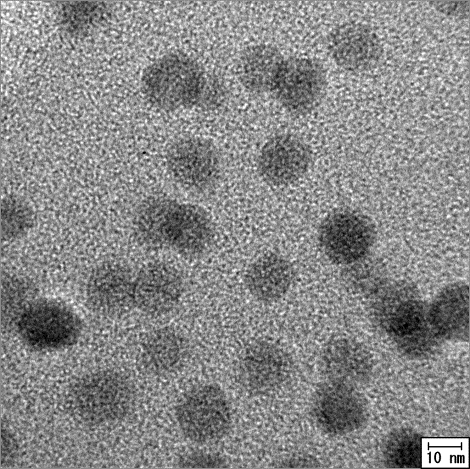

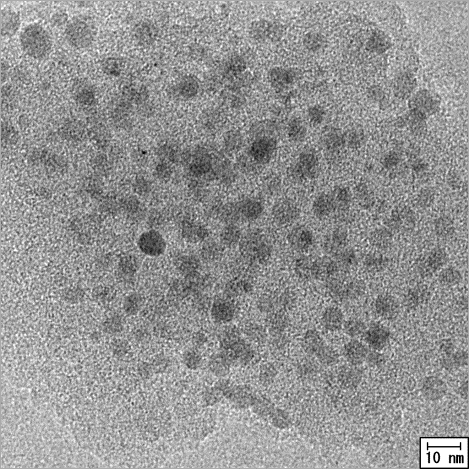

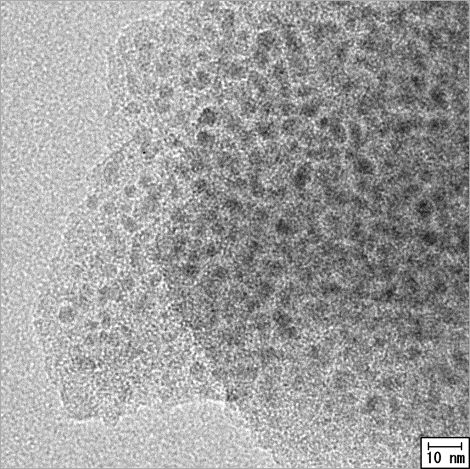


**a**

**b**

**c**

Figure S3 | Iron oxide nanoparticle sizes in the MCNC-SA carbon matrix. TEM images of MCNC-SA, prepared from iron(III) nitrate enneahydrate solutions with concentrations of (a) 1.25 g L^-1^, (b) 5.00 g L^-1^ and (c) 15.0 g L^-1^.


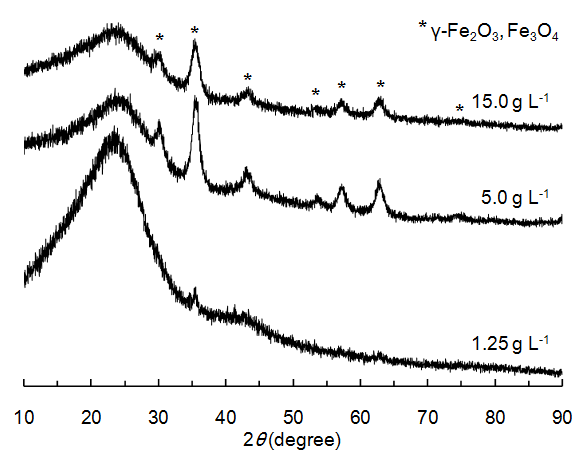

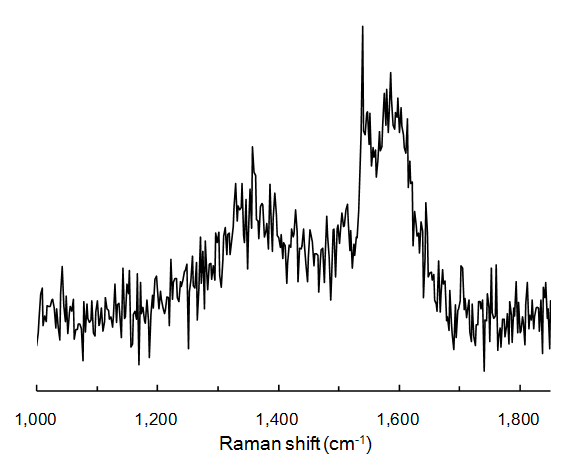


**a**

**b**

Figure S4 | Structures of MCNC-SA. (a) The XRD pattern of the samples. The diffraction peaks can be assigned to γ-Fe_2_O_3_ (JCPDS card No. 39-1346) or Fe_3_O_4_ (JCPDS card No. 19-0629). (b) Raman spectrum of the sample (concentration of iron(III) nitrate enneahydrate solution: 5.0 g L^-1^).

Table S3 | Mössbauer effect parameters of MCNC-SA (concentration of iron(III) nitrate enneahydrate solution: 5.0 g L^-1^).

| Temperature | Component | *δ* ^a)^ | *Δ* ^a)^ | *H* ^a)^ | %Fe |
| --- | --- | --- | --- | --- | --- |
| 293 K | [1] | +0.26 | 0.34 | 0 | 62 |
|  | [2] | +0.38 | 0.64 | 0 | 38 |
| 78 K | [1] | +0.46 | +0.03 | 506 | 18 |
|  | [2] | +0.57 | –0.11 | 434 | 45 |
|  | [3] | +0.53 | 0.61 | 0 | 37 |

1. *δ*: isomer shift (mm s^-1^), *Δ*: quadrupole shift (mm s^-1^), *H*: magnetic field (kOe).


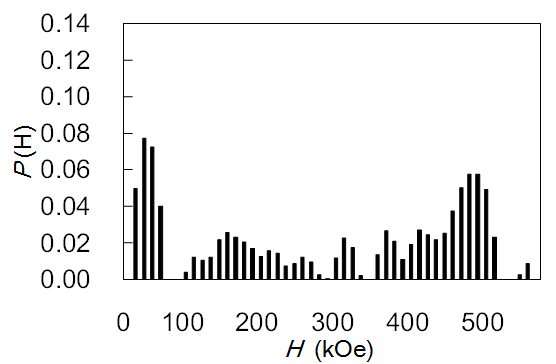


Figure S5 | The magnetic field distribution analysis for MCNC-SA at 78 K, assuming that the paramagnetic component gives a quadrupole shift doublet (concentration of iron(III) nitrate enneahydrate solution: 5.0 g L^-1^).


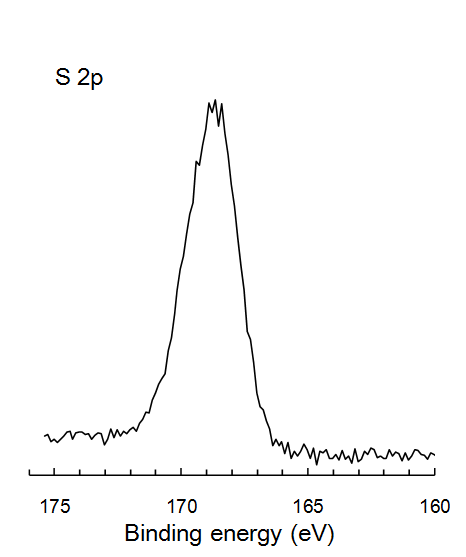

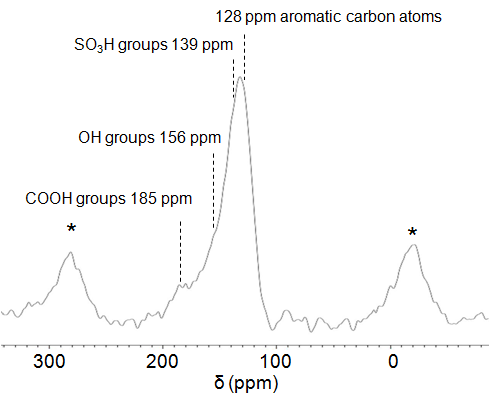


a

b

c

Figure S6 | Surface functional groups of MCNC-SA (concentration of iron(III) nitrate enneahydrate solution: 5.0 g L^-1^). (a) S 2p peak in the XPS spectrum. (b) ^13^C-DD/MAS NMR spectrum (* denotes spinning side bands). (c) FTIR spectrum.

Table S4 | Magnetic remanence (*M*_r_), saturation magnetization (*M*_s_), and coercivity (*H*_c_) of MCNC-SA samples synthesized using different iron nitrate concentrations.

| Samples  (with iron nitrate  concentration) | *M*_r_  (emu g^-1^) | *M*_s_  (emu g^-1^) | *H*_c_  (Oe) | *M*_r_ / *M*_s_ |
| --- | --- | --- | --- | --- |
| 1.25 g L^-1^ | 0.05 | 0.51 | 42 | 0.089 |
| 2.50 g L^-1^ | 0.05 | 1.61 | 25 | 0.028 |
| 5.00 g L^-1^ | 0.17 | 5.70 | 28 | 0.030 |
| 10.0 g L^-1^ | 0.20 | 8.38 | 23 | 0.024 |
| 15.0 g L^-1^ | 0.03 | 5.58 | 9 | 0.006 |


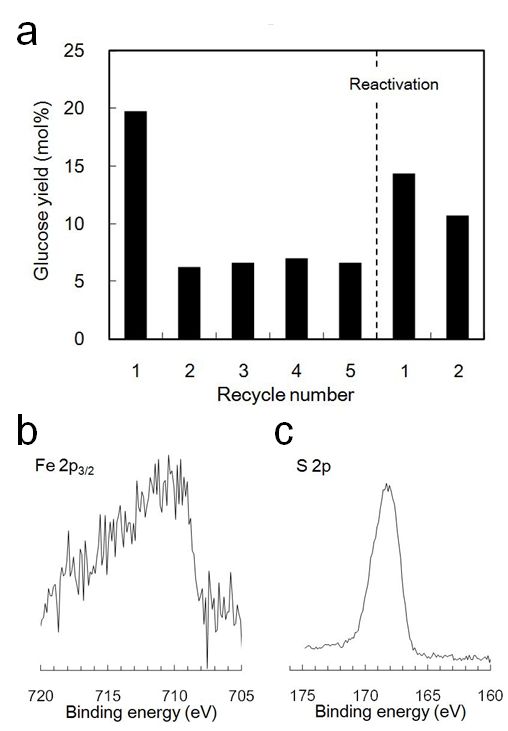


Figure S7 | Repeated hydrolysis of cellobiose using MCNC-SA (concentration of iron(III) nitrate enneahydrate solution: 1.25 g L^-1^). (a) Reaction conditions: MCNC-SA, 0.10 g; cellobiose, 0.12 g; water, 0.7 g; reaction temperature, 90 ºC; reaction time, 3 h. (b) Fe 2p_3/2_ and (c) C 2p XPS spectra of the catalyst after one use.
